# Expectancy Effects Cannot Be Neglected in MDMA-Assisted Therapy Research

**DOI:** 10.1021/acschemneuro.3c00692

**Published:** 2023-11-15

**Authors:** L. Jacob Flameling, Jacob S. Aday, Michiel van Elk

**Affiliations:** † Institute of Psychology, Leiden University, Leiden 2333 AK, The Netherlands; ‡ Department of Anesthesiology, University of Michigan, Ann Arbor, Michigan 48109, United States

**Keywords:** placebo-effects, MDMA-assisted psychotherapy, blinding, post-traumatic stress disorder

A recent paper by Mitchell et al. in *Nature Medicine* reported on the results from a study researching the potential use of 3,4-methylenedioxymethamphetamine-assisted therapy (MDMA-AT) for the treatment of posttraumatic stress disorder (PTSD).^1^ Although we applaud the efforts made to further establish the efficacy of MDMA-AT in the treatment of PTSD, we have some concerns regarding the discussion of potential expectancy effects in this trial. Expectancy effects typically occur when participants in a clinical trial identify which treatment arm they were assigned to and subsequently expect symptom alleviation if they are in the treatment group (i.e., placebo effects) or symptom worsening if they are in the control group (i.e., nocebo effects). Mitchell et al. put forth several arguments as to why they believed expectancy effects were minimal in this trial. Here, we explain why we find these arguments unconvincing. As most of the participants broke the blind, and participants’ expectations were not measured, it remains possible that expectancy effects may have contributed to the observed effects of MDMA-AT.

Mitchell et al. write that “several observations support expectancy mitigation in the current study”. Their first observation is that “prospective treatment expectancy would likely have been high in both study arms, with random assignment expected to distribute this equally between groups”. However, random assignment does not mitigate expectancy effects unless the blind is properly maintained.^2^ That is, if participants become aware of their treatment arm allocation, outcome expectancies regarding treatment efficacy can become a source of group differences. The blind was overwhelmingly broken by participants in this study, enhancing placebo effects in the treatment group and triggering possible nocebo effects in the control group. Hence, whereas pretreatment expectancies might have been comparable at the start of the study, post-treatment outcome expectancies likely differed dramatically between groups.

The authors next note that “the groups did not separate after the first experimental session”. We assume that the authors meant that the scores on the primary and secondary outcomes did not differ between the placebo and the treatment group after the first session and hence that no enhanced placebo response was observed in the MDMA-AT group compared to the control group. This rests on two assumptions: first, that unblinding happened after the first experimental session. However, it could well be that patients broke the blind only after the second or the third therapeutic session; we do not have data on this, as blinding quality was only assessed after study termination. The second assumption of the authors’ argument is that expectancy effects should become apparent after the first therapeutic session. However, it is possible that unblinded participants in the treatment group expected the treatment to be effective after multiple sessions, and thus expectancy effects may have fluctuated and changed in precision and strength over time.^3^ Again, we simply do not know, as expectancies were not measured.

The authors’ third observation is that “placebo with therapy dropouts did not uniformly occur after the first experimental session”. We point to Supplementary Table 3 of the paper, which shows that at least one participant in the control group dropped out after the second experimental session because they believed they were receiving placebo. Moreover, it should be noted that the difference in dropout rates between the treatment group (*N* = 1) and the placebo group (*N* = 8) was statistically significant at α = 0.05 (χ^2^(1) = 6.199, *p* = 0.01). This indicates that more participants dropped out in the placebo group, likely because of disappointment in the treatment. The observation that dropout did not occur after the first experimental session could be related to the fact that at that stage patients were still unsure whether they received a placebo or not. Indeed, given that the first dose (120 mg) was lower than the second and third doses (180 mg), it is possible that unblinding did not occur until later in the trial. Again, we do not know, as blinding quality and expectancies were not assessed over time.

Lastly, Mitchell et al. observe that “blinding survey data ... showed that not all participants correctly identified the treatment that they received”. However, 94% of participants in the treatment group and 75% in the placebo group guessed their treatment condition correctly. Had blinding been perfect, the percentages in both groups should have been approximately 50%. Alternatively, a majority of participants should have answered “cannot tell” when asked what treatment arm they believed to be in. Therefore, the fact that not *all* (i.e., 100%) of the participants guessed their treatment arm assignment correctly does not mean that expectancy effects were successfully mitigated.

In conclusion, we do not agree that the arguments put forward by Mitchell et al. support the claim that expectancy effects were successfully mitigated in this trial. We greatly commend the authors’ efforts to conduct and report on a blinding survey. We are also pleased to see that the authors measured the confidence participants had in their guess of which treatment arm they were in. This is an improvement compared to most studies in psychiatry as well as the first phase 3 study regarding MDMA-AT in the treatment of PTSD,^4^ which did not measure blinding quality. Yet, there is further room for improvement regarding the assessment of blinding quality and expectancy effects.

The simple message is measure more and report more! We need measurements of expectations and blinding not only at the end of the trial but throughout the different phases of the study. In recent years, the field of psychedelic science has suggested additional recommendations for addressing these issues that can be very easily implemented in any trial. For example, Muthukumaraswamy et al.^5^ recommend to assess the cause of unblinding, whether it is benign (i.e., due to positive treatment effects) or malicious (i.e., due to side-effects of the treatment). This recommendation was followed up upon by Szigeti et al.,^6^ who also developed the Correct Guess Rate Curve, which statistically adjusts for unblinding. Lastly, Muthukumaraswamy et al.^5^ and Aday et al.^2^ suggest to measure not only blinding quality but also the magnitude of expectancies, which has been successfully implemented in both micro- and macrodosing studies.^7−9^

Expectancy effects are ubiquitous, potent, and challenging to control for across different areas of medical research. Both the European Medical Agency and the U.S. Food and Drug Administration emphasize the challenge that unblinding and expectancy effects pose to the interpretation of psychedelic clinical trials in particular.^10,11^ We hope that the field of psychedelic research will take these recommendations seriously and that future studies by the Multidisciplinary Association for Psychedelic Studies and other institutions will make use of the most rigorous methodological tools available to assess blinding quality and expectancy effects.
